# Physico-chemical, sensory, and microbiological assessments of wheat-based biscuit improved with beniseed and unripe plantain

**DOI:** 10.1002/fsn3.135

**Published:** 2014-06-23

**Authors:** Helen Obioma Agu, Ndidiamaka Azuka Okoli

**Affiliations:** Food Science and Technology Department, Federal Polytechnic BauchiP.M.B. 0231, Bauchi, Bauchi State, Nigeria

**Keywords:** Biscuit, microbiological, physico-chemical, plant materials, sensory

## Abstract

The consumption of cereal foods such as biscuit has become very popular globally. Partial replacement of wheat flour with beniseed and unripe plantain flours rich in protein, vitamins, and minerals will increase nutrient, diversify utilization of beniseed and unripe plantain, and increase biscuit variety. Wheat composite biscuit was produced from wheat, beniseed, and unripe plantain flours. The composite flour was mixed in the proportion of 100:0:0, 80:10:10, 70:20:10, 60:30:10, and 50:40:10% of wheat, beniseed, and unripe plantain, respectively. The physical, sensory, chemical, and microbial properties of the biscuits were determined. The physical properties ranged from 6.80 g to 8.30 g for weight, spread ratio 6.93–7.38, and break strength 500–690 g. There was no significant difference (*P* < 0.05) in taste, crispness, flavor and texture of the biscuits while significant differences (*P* < 0.05) existed in color and overall acceptability. The proximate composition of the biscuits ranged from 1.84% to 2.55% for moisture, protein 8.03–9.26%, fat 30.07–35.81%, ash 2.94–3.68%, crude fiber 0.47–0.80%, carbohydrate 48.74–55.96%, and energy 526.53–554.21 kcal/100 g. The microbial count of the best biscuit after 20 days of storage was 4.0 × 10^3^ cfu/g for bacteria and mould contained 5.0 × 10^4^ cfu/g. This study forms a basis for new product development for the biscuit food industry.

## Introduction

Biscuit is a small thin crispy cake made from unleavened dough. Biscuits have been suggested as a better use of composite flour than bread due to their ready to eat form, wide consumption, relatively long shelf life, and good eating quality (Okpala and Chinyelu 2011). It may be regarded as a form of confectionery dried to very low moisture content (Okaka 1997). The dependence on the use of wheat flour is a major constraint in biscuit production. The recent shortage of wheat and the Federal government ban on its importation calls for research into alternative local sources of flour baking, for example, unripe plantain, beniseed, millet, and so on.

Wheat *(Triticum aestivum*) is a cereal grain grown all over the world for its highly nutritious and useful gain. It is one of the top three most produced crops in the world, along with corn and rice. According to Okaka (2005), only wheat contains substantial amount of gliadin and glutenin (special protein) which when kneaded with water give gluten, the elastic material important in yeast or aerated baked goods. In terms of total production tonnages used for food, it is currently second to rice as the main human food crop (Curtis et al. 2002). Much of the carbohydrate fraction of wheat is starch. Wheat starch is an important commercial product of wheat, but second in economic value to wheat gluten. The principal parts of wheat flour are gluten and starch (International Starch Institute 2008).

Beniseed commonly known as sesame seed and botanically *Sesamum indicum* is one of the cultivated oil seed crops grown all over the world. The beniseed flour can be used in food products as a protein, tryptophan, and methionine supplement (Escamilla-Silva et al. 2003). It is precisely these amino acids that distinguished it from other oil seeds. Beniseed is an excellent source of high-quality oil (very stable and free-flavor component and having natural anti-oxidant which prevent aging) and vital for the production of liver cells (Weiss 2000). Beniseed oil is a natural salad oil requiring little or no winterization and is one of the few vegetable oils that can be used directly without refining (Sudhir et al. 1996, Gandhi 1998). The seed cake is a good source of protein supplement in the animal food industry.

Plantain is the common name for herbaceous plants of the genus *Musa*. Plantains are classified formally as *Musa acuminate* and *Musa balbisiana* depending on their genomic constitution. Plantain provides more than 25% of the carbohydrate requirements for over 70 million people. Plantains tend to be firmer and lower in sugar content. They are commonly cooked or otherwise processed and are used either when green or unripe (and therefore starchy) or over ripe (and therefore sweet) (Oke et al. 1998). An average plantain has about 220 calories and is a good source of potassium and dietary fiber (Randy et al. 2007). It is rich in carbohydrate, dietary fiber, irons, vitamins, and minerals. This nutritious food is ideal for diabetics, children, and pregnant women. It can also be a good supplement for marasmus patients. Plantain contains small amount of serotonin which has the ability to dilate the arteries and improve blood circulation. Its regular consumption helps to cure anemia (low blood level) and maintain a healthy heart (USDA Nutrient Database 2010). A diet of unripe plantain is filling and can also be a good inclusion in a weight loss diet plan (Oke et al. 1998).

Today, plant protein play significant roles in human nutrition particularly in developing countries where average protein intake is less than required. Due to inadequate supplies of animal proteins, there has been a constant search for new protein sources, for use as both functional food ingredients and nutritional supplements (Obizoba and Anyike 1994). An example is Beniseed.

Plant protein products are gaining increased interest as ingredients in food systems throughout many parts of the world. The final success of utilizing plant protein and carbohydrate as additives depends greatly upon the favorable characteristics that they impart on foods. The development and use of defatted beniseed flour and unripe plantain flour would provide industry with new high protein and iron food ingredients for product formulation and protein fortification (Uwala 2002).

Beniseed, unripe plantain–wheat-based biscuit could be made by the addition of unripe plantain and beniseed flour to wheat flour with condiments such as fat, baking powder as a leavening agent, egg as a stabilizer, and milk as a tenderizer (Escamilla-Silva et al. 2003). Despite the inherent potentials of beniseed and unripe plantain, little research has been carried out to incorporate them in most food formulations. The aims and objectives of this research work were (1) to produce biscuits from wheat, beniseed, and unripe plantain flours. (2) to evaluate the physical properties of the biscuits. (3) to determine the acceptability of the biscuits using sensory evaluation. (4) to evaluate the chemical composition of the best three biscuits and microbiological properties of the best biscuit.

## Materials and Methods

### Source of materials

The consumable materials were purchased from Muda Lawal market, Bauchi, Bauchi State and they are as follows: wheat flour (Honey well, Lagos, Nigeria), beniseed (*Sesamum indicum*), unripe plantain (*Musa paradisiaca),* fat (Simas, Jakarta, Indonesia), salt (Royal salt Ltd, Abuja, Nigeria), eggs, and baking powder (Princess K, Kano, Nigeria). The equipment and chemicals were of analytical grade.

### Production of beniseed flour

**Figure 1:**
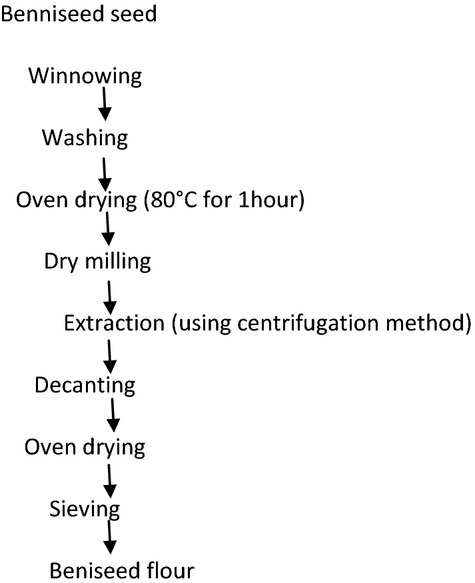
Flow chart of the production process of beniseed flour.

Beniseed flour was produced using Anuonye et al. (1999) method as shown in Figure 1. The method involved winnowing, washing, and oven dried (Prolabo, Paris, France; No. 53867 V220) at 80°C for 1 h to reduce the moisture content of the seeds and to aid easy milling. Oil was extracted using ethanol by centrifugation (Prolabo, No. 83175 V220) method. The flour was oven dried to remove the residual ethanol and sieved using 0.315 mm mesh screen to obtain a uniform particle size of flour.

**Table 1:** Recipe for biscuit samples.

Wheat flour (%)	Beniseed flour (%)	Unripe plantain flour (%)	Fat (g)	Egg (g)	Sugar (g)	Salt (g)	Baking powder (g)
100	0	0	45	30	55	0.6	3.6
80	10	10	45	30	55	0.6	3.6
70	20	10	45	30	55	0.6	3.6
60	30	10	45	30	55	0.6	3.6
50	40	10	45	30	55	0.6	3.6

### Production of unripe plantain flour

**Figure 2:**
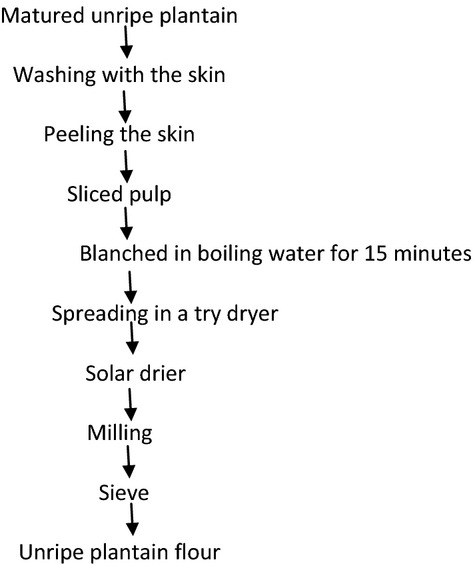
Flow chart for production process of unripe plantain flour.

The unripe plantain was processed using Okaka (1997) method as shown in Figure 2. The unripe plantain was washed along with the skin to reduce contamination, peeled manually, sliced to obtain a uniform size for easy drying, blanched using Steam blancher (Stott Benham Ltd, Halifax, West Yorkshire, U.K., E56) for 15 min as to prevent color change, dried using solar energy, and ground using manual hand engine. The flour was sieved to obtain uniform particle size using 0.315 mm mesh screen.

**Table 2:** Physical analysis of biscuit samples.

Wheat flour (%)	Beniseed flour (%)	Unripe plantain flour (%)	Weight (g)	Spread ratio	Break strength (g)
100	0	0	8.10	7.00	690
80	10	10	8.30	7.15	650
70	20	10	7.80	7.15	630
60	30	10	7.70	7.38	590
50	40	10	6.80	6.93	500

### Production process of wheat, beniseed, unripe plantain composite biscuit

**Figure 3:**
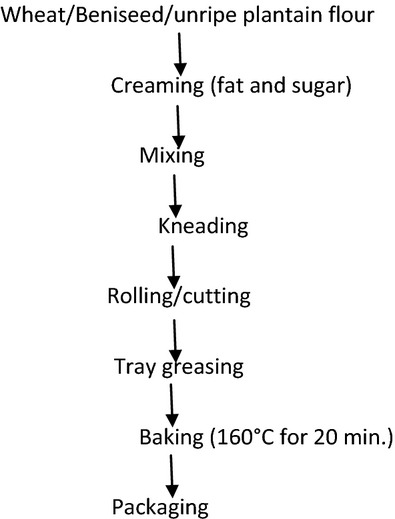
Flow chart for production process of wheat, beniseed, unripe plantain composite biscuit.

Five value added biscuit products were produced from three different raw materials, namely wheat, beniseed, and unripe plantain, at different proportions of 100:0:0%, 80:10:10%, 70:20:10%, 60:30:10%, and 50:40:10%, respectively as shown in Table 1. The basic formulation was flour (100 g), fat (45 g), egg (30 g), sugar (55 g), salt (0.6 g), and baking powder (3.6 g). The biscuits were produced using Okaka (1997) method ( Fig. 3).

### Physical analysis

#### Spread ratio

Two rows of five well-formed biscuits were made and the height was measured. They were also arranged horizontally edge to edge and the sum of the diameters was measured. The spread ratio was calculated as diameter divided by height (Gomez et al. 1997).

#### Break strength

Okaka and Isieh (1997) method was used. Biscuit of known thickness (0.4 cm) was placed centrally between two parallel metal bars (3 cm apart). The weights were added on the biscuit until the biscuit snapped. The least weight that caused the breaking of the biscuit was regarded as the break strength of the biscuit.

### Sensory evaluation of the biscuit sample

The sensory evaluation of the samples was carried out using 20 panelists from Federal Polytechnic, Bauchi, Bauchi State. A nine-point Hedonic scale one (1) and nine (9) representing “extremely dislike” and “extremely like”, respectively, was used. The qualities assessed include color, texture, taste, flavor, crispness, and general acceptability. Coded samples of the same size and temperature (29 ± 3°C) were served on clean white plates to judges in different boots under the florescent light. Only one sensory attribute was tasted at one sitting.

### Proximate composition

The moisture, protein, fat, ash, and crude fiber contents were determined by the AOAC (2000). The total carbohydrate was determined by difference: Carbohydrate = 100 − (% moisture + % protein + % fat + % ash + % crude fiber). Energy content was determined as described by Marero et al. (1988).

### Microbiological analysis

The pour plate method of knowing and counting the number of viable bacteria present in the sample was adopted as described by Jideani and Jideani (2006). Two grams of the sample was weighed out for analysis and evaluated. Quarter strength of peptone water solution was prepared by dissolving 3 g in 200 mL of distilled water. Nutrient Agar (NA) was prepared by dissolving 4.6 g in 200 mL of distilled water; serial dilution was carried out on the biscuit samples and plating of the sample was performed.

### Statistical analysis

The various results obtained were subjected to Analysis of Variance (ANOVA) and Duncan (1955) Multiple range test was used to separate means where significant differences existed.

## Results and Discussion

Physical analysis of the biscuit is shown in Table 2. The weight of the biscuit samples decreased with increase in beniseed flour with 80:10:10% wheat, beniseed, and unripe plantain flour biscuit having the highest weight and 50:40:10% wheat, beniseed, and unripe plantain flour biscuit having the lowest. This decrease in weight may be as a result of addition of beniseed and unripe plantain flour as both flours are lighter in weight when compared to wheat flour (Okeagu 2001).

The spread ratio of the biscuit increased from 7.00 to 7.38. The increase is an indication of the binding properties of the flour and of the texture of the biscuits. This increase may be as a result of increase in beniseed flour as both unripe plantain and beniseed flour has poor binding properties.

The break strength value ranged from 500 g to 690 g with sample 80:10:10% wheat, beniseed, and unripe plantain flour having the highest and sample 50:40:10% wheat, beniseed and unripe plantain flour having the lowest. This may be due to poor quality of beniseed and unripe plantain flour to form a strong network in both flours in a molten state at baking temperature.

The mean scores for the sensory evaluation of the biscuits are shown in Table 3. There was no significant difference (*P* < 0.05) in taste, crispness, flavor, and texture of the biscuits while significant differences (*P* < 0.05) existed in color and overall acceptability. Sample AMA (80:10:10% wheat, beniseed, and unripe plantain flour biscuit) had the highest mean value of 6.85 when compared to 7.30 for 100% wheat flour biscuit. Sample OKO (50:40:10% wheat, beniseed, and unripe plantain flour biscuit) had the lowest mean value of 5.50. This may be as a result of addition of beniseed and unripe plantain flour. This showed that sample AMA (80:10:10% wheat, beniseed, and unripe plantain flour biscuit) compared favorably with the 100% wheat flour biscuit followed by sample MAK (70:20:10% wheat, beniseed, and unripe plantain flour biscuit) and then sample AKA (60:30:10% wheat, beniseed, and unripe plantain flour biscuit). There was a general decrease in the acceptability of the biscuits with increase in beniseed flour.

**Table 3:** Sensory evaluation of the biscuit samples.

Sample code	Wheat flour (%)	Beniseed flour (%)	Unripe plantain flour (%)	Taste	Crispness	Flavor	Texture	Colour	Overall acceptability
IDA	100	0	0	7.30^a^ ± 1.72	6.85^a^ ± 2.21	6.55^a^ ± 1.50	6.80^a^ ± 1.70	7.30^a^ ± 1.45	8.00^a^ ± 0.86
AMA	80	10	10	6.80^a^ ± 1.54	6.85^a^ ± 1.88	6.50^a^ ± 1.60	6.35^a^ ± 1.84	6.85^ab^ ± 1.76	7.50^ab^ ± 1.79
MAK	70	20	10	6.55^a^ ± 2.01	6.55^a^ ± 1.85	6.30^a^ ± 1.75	6.30^a^ ± 1.56	6.60^ab^ ± 2.24	6.90^b^ ± 1.59
AKA	60	30	10	6.55^a^ ± 2.19	6.55^a^ ± 1.14	6.25^a^ ± 1.75	6.00^a^ ± 1.69	6.15^ab^ ± 1.85	6.80^b^ ± 1.79
OKO	50	40	10	6.20^a^ ± 1.73	6.55^a^ ± 1.23	5.59^a^ ± 1.70	5.80^a^ ± 2.12	5.55^b^ ± 1.90	6.40^b^ ± 1.79

Values are mean ± standard deviation of 20 panelists. Means within each column not followed by the same superscript are significantly different (*P* < 0.05) from each other using Duncan multiple range test.

The proximate composition of the best three biscuit samples, beniseed flour, and 10% unripe plantain are shown in Table 4. The moisture content of the biscuits decreased from 2.55% to 1.84%. This could be due to increase in protein content as a result of defatted beniseed flour and protein has more affinity to moisture than carbohydrate (Okeagu 2001). This could be an advantage to keep quality (shelf life) as most spoilage organisms may not be able to survive. This finding agreed with work carried out on “effect of sesame seed flour on millet biscuit characteristics (Alobo 2001).

**Table 4:** Proximate composition of biscuit samples.

Materials/nutrients	Samples		
	AMA	MAK	AKA
Wheat flour (%)	80	70	60
Beniseed flour (%)	10	20	30
Unripe plantain flour (%)	10	10	10
Moisture (%)	2.55 ± 0.28	2.01 ± 0.11	1.84 ± 0.17
Protein (%)	8.03 ± 0.06	8.32 ± 0.19	9.26 ± 0.42
Fat (%)	30.07 ± 0.48	33.25 ± 0.23	35.81 ± 1.12
Ash (%)	2.94 ± 0.16	3.16 ± 0.03	3.68 ± 0.18
Crude fiber (%)	0.47 ± 0.08	0.80 ± 0.04	0.69 ± 0.04
Carbohydrate (%)	55.96 ± 0.09	52.46 ± 0.60	48.74 ± 1.59
Energy (kcal/100 g)	526.53 ± 4.53	542.39 ± 0.42	554.21 ± 5.42

Values are mean ± standard deviation of duplicate samples.

The protein content of the biscuits increased from 8.03% to 9.26% with increase in the percentage of beniseed flour. This was due to the addition of the defatted beniseed flour which is a good source of protein supplement. The fat content increased from 30.07% to 35.81% with sample AMA (80:10:10% wheat, beniseed, and unripe plantain flours) having the highest. This increase may be due to the addition of beniseed flour which is a good source of oil (Olayanju et al. 2006).

The ash content ranged from 2.94% to 3.68% with increase in beniseed flour and unripe plantain flour. Plantain flour is a good source of iron and other mineral contents (Gandhi 1998). The crude fiber content increased from 0.47% to 0.80%. This was due to added beniseed and unripe plantain flour which are good sources of fiber content. This is an advantage as it helps in bowel movement and easy digestibility. The carbohydrate content decreased while the energy content increased with increase in beniseed and unripe plantain flour. This showed that the biscuits have good energy content.

The microbial count of the best biscuit sample (80:10:10% wheat, beniseed and unripe plantain flours) after 20 days of storage in polyethylene pack with paperboard is shown in Table 5. It was observed that there was no growth in the first 4 days. On the 20th day of storage, the bacteria count was 4.0 × 10 cfu/g while the mould count was 5.0 × 10^4^ cfu/g. The growth observed could be due to post-processing contamination.

**Table 5:** Microbial analysis of 80% wheat flour, 10% beniseed flour, and 10% unripe plantain flour biscuit.

Sample	Days	Bacterial count (cfu/g)	Mould count (cfu/g)
80% wheat flour, 10% beniseed flour, and 10% unripe plantain flour biscuit	4	Nil	Nil
	8	1.0 × 10^3^	2.0 × 10^4^
	12	2.0 × 10^3^	3.0 × 10^4^
	20	4.0 × 10^3^	5.0 × 10^4^

Cfu/g, colony-forming unit per gram.

## Conclusion

An acceptable biscuit was formulated from wheat flour, beniseed flour, and unripe plantain flour. The composition of beniseed flour and unripe plantain made them dietary items. Analysis carried out showed that the formulated biscuit is rich in protein, carbohydrate, ash, and energy. The formulation of the biscuit has provided another means of utilizing our abundant beniseed seed and unripe plantain fruit. The biscuit produced has low microbial load and therefore has a long shelf life.

## Supporting Information

**Table S1.** Sensory evaluation of the biscuit samples. **Table S2.** Proximate composition of biscuit samples. **Table S3.** Microbial analysis of 80% wheat flour, 10% beniseed flour, and 10% unripe plantain flour biscuit.
