# Supplementary material for: Physico-chemical, sensory, and microbiological assessments of wheat-based biscuit improved with beniseed and unripe plantain
Source: Food Sci Nutr. 2014 Jun 23;2(5):464–9. doi: 10.1002/fsn3.135 (PMC4237476; doi:10.1002/fsn3.135)
Supplement: Supplementary file 1 [file fsn30002-0464-sd1.docx]

**Table S1. Sensory Evaluation of the Biscuit Samples**

| **Sample code** | **Wheat flour (%)** | **Benniseed flour (%)** | **Unripe plantain flour (%)** | **Taste** | **Crispness** | **Flavour** | **Texture** | **Colour** | **Overall Acceptability** |
| --- | --- | --- | --- | --- | --- | --- | --- | --- | --- |
| IDA | 100 | 0 | 0 | 7.30^a^ ± 1.72 | 6.85^a^ ± 2.21 | 6.55^a^ ± 1.50 | 6.80^a^ ± 1.70 | 7.30^a^ ± 1.45 | 8.00^a^ ± 0.86 |
| AMA | 80 | 10 | 10 | 6.80^a^ ± 1.54 | 6.85^a^ ± 1.88 | 6.50^a^ ± 1.60 | 6.35^a^ ± 1.84 | 6.85^ab^ ± 1.76 | 7.50^ab^ ± 1.79 |
| MAK | 70 | 20 | 10 | 6.55^a^ ± 2.01 | 6.55^a^ ± 1.85 | 6.30^a^ ± 1.75 | 6.30^a^ ± 1.56 | 6.60^ab^ ± 2.24 | 6.90^b^ ± 1.59 |
| AKA | 60 | 30 | 10 | 6.55^a^ ± 2.19 | 6.55^a^ ± 1.14 | 6.25^a^ ± 1.75 | 6.00^a^ ± 1.69 | 6.15^ab^ ± 1.85 | 6.80^b^ ± 1.79 |
| OKO | 50 | 40 | 10 | 6.20^a^ ± 1.73 | 6.55^a^ ± 1.23 | 5.59^a^ ± 1.70 | 5.80^a^ ± 2.12 | 5.55^b^ ± 1.90 | 6.40^b^ ± 1.79 |

Values are mean ± standard deviation of 20 panelists. Means within each column not followed by the same superscript are significantly different (p < 0.05) from each other using Duncan multiple range test.

.

**Table S2. Proximate composition of Biscuit samples**

|  | **Samples** | | |
| --- | --- | --- | --- |
| **Materials/Nutrients** | **AMA** | **MAK** | **AKA** |
| Wheat flour (%) | 80 | 70 | 60 |
| Beniseed flour (%) | 10 | 20 | 30 |
| Unripe plantain flour (%) | 10 | 10 | 10 |
| Moisture (%) | 2.55 ± 0.28 | 2.01 ± 0.11 | 1.84 ± 0.17 |
| Protein (%) | 8.03 ± 0.06 | 8.32 ± 0.19 | 9.26 ± 0.42 |
| Fat (%) | 30.07 ± 0.48 | 33.25 ± 0.23 | 35.81 ± 1.12 |
| Ash (%) | 2.94 ± 0.16 | 3.16 ± 0.03 | 3.68 ± 0.18 |
| Crude Fibre (%) | 0.47 ± 0.08 | 0.80 ± 0.04 | 0.69 ± 0.04 |
| Carbohydrate (%) | 55.96 ± 0.09 | 52.46 ± 0.60 | 48.74 ± 1.59 |
| Energy (Kcal/100g) | 526.53 ± 4.53 | 542.39 ± 0.42 | 554.21 ± 5.42 |

Values are mean ± standard deviation of duplicate samples.

**Table S3. Microbial analysis of 80% wheat flour, 10% beniseed flour, and 10% unripe plantain flour biscuit**.

| **Sample** | **Days** | **Bacterial count (cfu/g)** | **Mould count**  **(cfu/g)** |
| --- | --- | --- | --- |
| 80% wheat flour, 10% beniseed flour, and 10% unripe plantain flour biscuit. | 4 | Nil | Nil |
|  | 8 | 1.0 x 10^3^ | 2.0 x 10^4^ |
|  | 12 | 2.0 x 10^3^ | 3.0 x 10^4^ |
|  | 20 | 4.0 x 10^3^ | 5.0 x 10^4^ |

Cfu/g = colony forming unit per gram
